# Identification and Development of Subtypes With Poor Prognosis in Pan-Gynecological Cancer Based on Gene Expression in the Glycolysis-Cholesterol Synthesis Axis

**DOI:** 10.3389/fonc.2021.636565

**Published:** 2021-03-24

**Authors:** Guangwei Wang, Xiaofei Liu, Dandan Wang, Meige Sun, Qing Yang

**Affiliations:** ^1^Department of Obstetrics and Gynecology, Shengjing Hospital of China Medical University, Shenyang, China; ^2^Department of Obstetrics and Gynecology, Shenyang Women's and Children's Hospital, Shenyang, China

**Keywords:** glycolysis, gynecological pan-cancer, cholesterol, immune infiltration, prognosis

## Abstract

**Objective:** Metabolic reprogramming is an important biomarker of cancer. Metabolic adaptation driven by oncogenes allows tumor cells to survive and grow in a complex tumor microenvironment. The heterogeneity of tumor metabolism is related to survival time, somatic cell-driven gene mutations, and tumor subtypes. Using the heterogeneity of different metabolic pathways for the classification of gynecological pan-cancer is of great significance for clinical decision-making and prognosis prediction.

**Methods:** RNA sequencing data for patients with ovarian, cervical, and endometrial cancer were downloaded from The Cancer Genome Atlas database. Genes related to glycolysis and cholesterol were extracted and clustered coherently by using *ConsensusClusterPlus*. The mutations and copy number variations in different subtypes were compared, and the immune scores of the samples were evaluated. The *limma* R package was used to identify differentially expressed genes between subtypes, and the *WebGestaltR* package (V0.4.2) was used to conduct Kyoto Encyclopedia of Genes and Genomes pathway and Gene Ontology functional enrichment analyses. A risk score model was constructed based on multivariate Cox analysis. Prognostic classification efficiency was analyzed by using *timeROC*, and internal and external cohorts were used to verify the robustness of the model.

**Results:** Based on the expression of 11 glycolysis-related genes and seven cholesterol-related genes, 1,204 samples were divided into four metabolic subtypes (quiescent, glycolysis, cholesterol, and mixed). Immune infiltration scores showed significant differences among the four subtypes. Survival analysis showed that the prognosis of the cholesterol subtype was better than that of the quiescent subtype. A nine-gene signature was constructed based on differentially expressed genes between the cholesterol and quiescent subtypes, and it was validated by using an independent cohort of the International Cancer Genome Consortium. Compared with existing models, our nine-gene signature had good prediction performance.

**Conclusion:** The metabolic classification of gynecological pan-cancer based on metabolic reprogramming may provide an important basis for clinicians to choose treatment options, predict treatment resistance, and predict patients' clinical outcomes.

## Background

Ovarian cancer, cervical cancer, and endometrial cancer are the most common cancers of the female reproductive system. Ovarian cancer is the deadliest, killing about 150,000 women each year (1). Due to a lack of typical clinical symptoms in early ovarian cancer, 75% of ovarian cancer patients are diagnosed with advanced cancer, and more than 70% relapse after treatment (2). The survival rate of ovarian cancer patients in most countries, which is about 30–50%, has not changed much in the past 20 years (3). In 2019, the number of new endometrial cancer cases in the United States was 61,880, and the number of deaths due to endometrial cancer was 12,160 (4). Among these gynecological cancers, its mortality rate is second only to ovarian cancer. Eighty percent of cervical cancer cases occur in developing countries, and there are about 570,000 new cases and 311,000 deaths per year worldwide (5). Distant metastasis is present in at least 59% of ovarian cancer patients, 15% of cervical cancer patients, and 9% of endometrial cancer patients (6–8). Therefore, seeking a reliable early diagnostic index and potential effective therapeutic targets is the best strategy to conquer gynecological malignant tumors.

Abnormal cell metabolism is an important feature of malignant tumors (9, 10). On one hand, oncogene activation or tumor suppressor gene inactivation, the tumor microenvironment, and metabolic gene mutations lead to the metabolic adaptation of tumor cells to meet the energy supply and macromolecular synthesis needs to maintain malignant biological behavior. On the other hand, abnormal cell metabolism can be used as an upstream event to drive the occurrence and development of tumors, and metabolism-related proteins and metabolites can affect tumor-related signal transduction and malignant biological behavior (11). As early as the early 20th century, Otto Warburg confirmed that even under the condition of sufficient oxygen, tumor cells metabolize glucose by glycolysis. This characteristic mode of metabolism by tumor cells is named the Warburg effect, or glycolysis (12). Glycolysis not only meets tumor cells' rapid growth and proliferation needs for ATP, macromolecular raw materials, and NADH/NADPH reduction equivalents, but it also provides an ideal target for tumor drug development and therapy (13). At the same time, tumor cells show high affinity for cholesterol, which not only influences the synthesis of cholesterol from scratch, but also the uptake and outflow of cholesterol, leading to reconnection of the cholesterol homeostasis pathway. However, like gene heterogeneity, the metabolism of tumor cells is highly heterogeneous. That is, there is no single universal change in tumor cell metabolism. Moreover, tumor cells produce different genetic variations during their occurrence, development, and treatment. Pan-cancer analysis of global metabolic pathways has shown that tumor metabolic heterogeneity is associated with survival, somatic cell-driven gene mutations, and tumor subtypes, but whether the heterogeneity of different metabolic pathways can be used to classify gynecological small tumors into clinically relevant subtypes has not been studied.

Based on glycolysis- and cholesterol-related gene expression patterns, we divided gynecological pan-cancer (ovarian, endometrial, and cervical) into four subtypes in order to further analyze differences in survival time, molecular mutations, and other clinical features among different metabolic subtypes. In this study, we proposed a clinically feasible classification scheme for gynecological pan-cancer. Our metabolic subtypes may provide a new approach for prognosis prediction and targeted therapy of gynecological pan-cancer.

## Methods and Materials

### Sources and Preprocessing of Data

RNA sequencing (RNA-Seq) expression data, single nucleotide variant (SNV)/InDel mutation data, copy number variation (CNV) data, and clinical follow-up information of ovarian cancer, cervical cancer, and endometrial cancer were downloaded from The Cancer Genome Atlas (TCGA) database. RNA-Seq data and clinical follow-up information of ovarian cancer were also downloaded from the International Cancer Genome Consortium (ICGC) database. Glycolysis- and cholesterol-related genes were derived from REACTOME_GLYCOLYSIS (29 genes) and REACTOME_CHOLESTEROL_BIOSYNTHESIS (24 genes) from the c2.cp.reactome.v6.2.symbols.gm files of the MSigDB database, for a total of 53 genes.

The RNA-Seq data of the TCGA database were processed according to the following steps: (1) retain the expression spectrum of the primary solid tumor sample; (2) convert the ensemble into the gene symbol; (3) take the median value of the expression of multiple gene symbols; and (4) convert the expression spectrum from FPKM to TPM and perform log2 transformation.

The following steps were performed for the ICGC database: (1) remove normal tissue samples; and (2) remove samples with no clinical follow-up information. After data preprocessing, 91 ICGC samples were obtained (Supplementary Table 1), in addition to 372 TCGA-OV samples, 291 TCGA-CESC samples, and 541 TCGA-UCEC samples, for a total of 1,204 TCGA samples (Supplementary Table 2). The batch effect was eliminated by using the *ComBat* function in the R package, and principal component analysis was used to analyze the data before and after the elimination of the batch effect (Supplementary Figure 1).

### Identification of Molecular Subtypes of Gynecological Pan-Cancer

A total of 46 genes, including 25 glycolysis-related genes and 21 cholesterol-related genes, were obtained after the TCGA expression profile data were filtered by removing genes with zero expression in all samples (Supplementary Table 3). *ConsensusClusterPlus* V1.48.0 (parameters: reps = 100, pitem = 0.8, pfeature = 1, distance = “Spearman”) was used to cluster glycolysis- and cholesterol-related genes. The Z-score was used to classify the TCGA dataset (*n* = 1,204) using the median values of glycolysis- and cholesterol-related gene expression.

### Analysis of *MPC1/2* Expression

To identify the genes positively and negatively related to *MPC1/2* expression, the correlations between *MPC1/2* and all other detected genes were analyzed. The Spearman correlation coefficients and corresponding *P-*values between *MPC1/2* and other genes were calculated, and the false discovery rate (FDR) was calculated by using the Beyer-Hardwick method. After filtering, 961 (Supplementary Table 4) and 1,143 genes (Supplementary Table 5) were positively and negatively correlated with *MPC1/2* expression, respectively (Spearman correlation, FDR < 0.05).

To further explore the enrichment pathways of genes with positive and negative correlations with *MPC1/2* expression, Gene Ontology (GO) enrichment analysis of the genes with positive and negative correlations with *MPC1/2* expression was performed by using the *WebGestaltR* package (V0.4.2) in R (FDR < 0.05).

### Identification of Differentially Expressed Genes and Functional Enrichment

The differentially expressed genes (DEGs) between cholesterol subtype and quiescent subtype were calculated by using the *limma* R package (14) and filtered according to the threshold: FDR < 0.05 and | FC | > 1.2. Then, the DEGs were selected for functional enrichment by using *Goplot* package.

### Construction and Evaluation of Prognostic Risk Model Based on Differentially Expressed Genes

#### Random Grouping of Training Dataset Samples

The 372 ovarian cancer samples with expression spectrum data in the TCGA database were divided into the training cohort and verification cohort. To avoid the influence of random assignment bias on the stability of subsequent modeling, all samples were randomly grouped 100 times before being put back, and grouping was carried out according to the training cohort-to-verification cohort ratio of 1:1. The most suitable training and verification cohorts were selected according to the following conditions: (1) the two groups were similar in age distribution, follow-up time, and patient death ratio; and (2) the sample sizes of the two groups were close to each other after gene expression profile clustering.

#### Univariate and Multivariate Cox Analyses of Training Cohort Samples

In the training cohort, univariate Cox proportional hazard regression was carried out by using the survival *coxph* function, and *p* < 0.01 was selected as the threshold value. To reduce the number of genes, we used the Akaike information criterion (AIC) algorithm to analyze the genes. The step AIC method in the MASS package starts from the most complex model and removes one variable to reduce the AIC; the smaller the value is, the better the result is, and the more superior the model is, meaning that the fitting degree of the model is better with fewer parameters. Nine differential genes were obtained.

#### Validation of Risk Score Model

The robustness of the model was further verified by using internal datasets (TCGA validation sets and all datasets) and external datasets (ICGC datasets). Using the same model and the same coefficients as the training set, the risk score of each sample was calculated according to the expression of the sample, and the risk score distribution of the sample was plotted. Receiver operating characteristic (ROC) analysis of risk score prognostic classification was performed by using *timeROC*, and the predictive classification efficiency was calculated for 1, 3, and 5 years.

#### Gene Enrichment Analysis

Gene set enrichment analysis was carried out by using the *GSVA* software package in R to calculate the scores of different functions for each sample, then the single-sample gene set enrichment analysis scores of each function for each sample were obtained. Features with a correlation greater than 0.35 were selected.

## Results

### Analysis of Glycolysis- and Cholesterol-Related Gene Expression to Identify Four Subtypes of Gynecological Pan-Cancer

**Figure 1:**
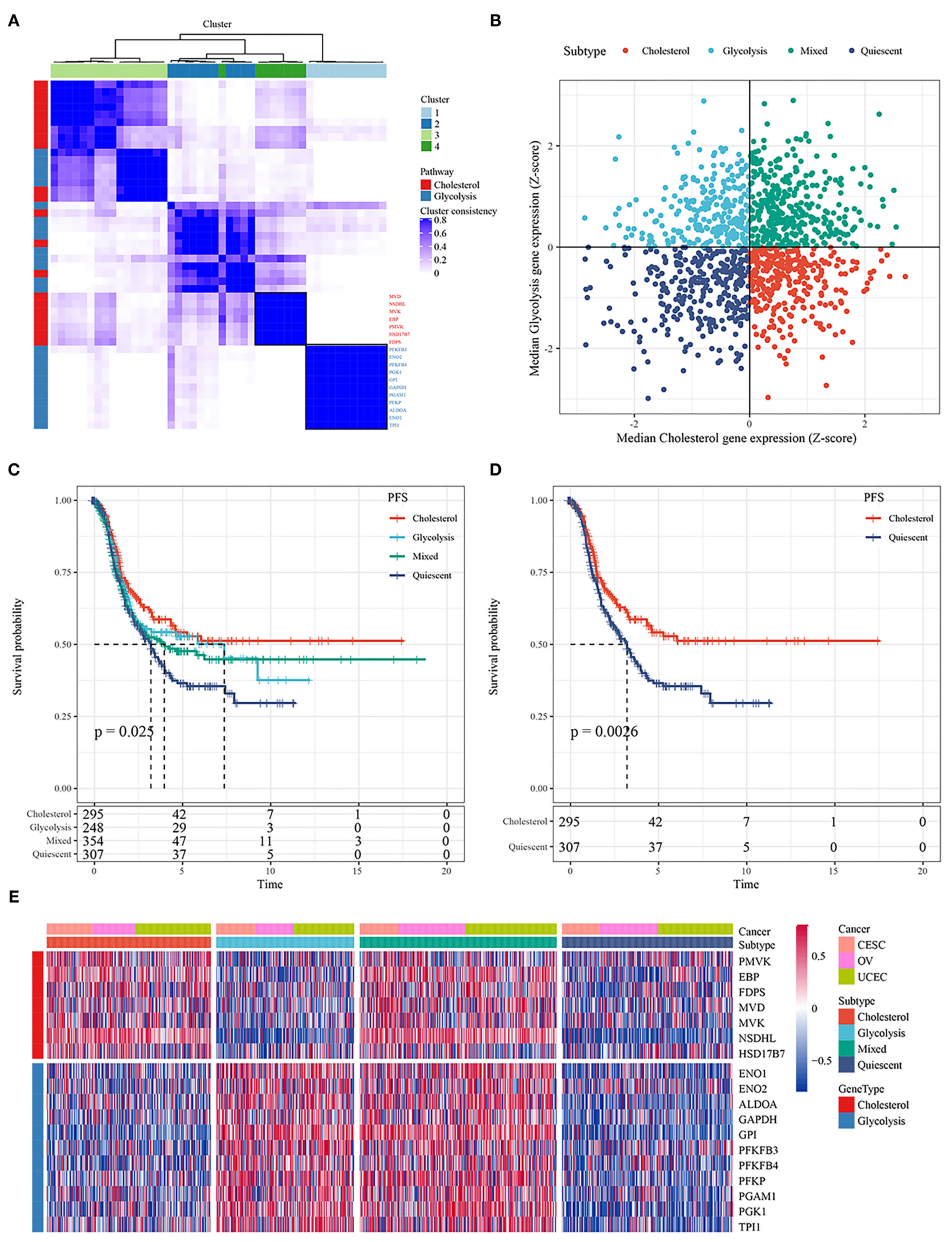
**(A)** Consistent clustering of glycolysis and cholesterol genes; **(B)** Classification of samples according to glycolysis and cholesterol gene expression; **(C)** PFS curve of four molecular subtypes in TCGA ovarian cancer samples; **(D)** PFS curve between cholesterol and quiescent subtype; **(E)** Cluster heatmaps of 18 related genes.

The RNA-seq data for ovarian, cervical, and endometrial cancer in the TCGA database were integrated, and a total of 1,204 samples were obtained for analysis after all samples were stripped of batch effects. Under K = 4, glycolysis- and cholesterol-related genes were clustered by using consensus clustering (Figure 1A). Based on the median expression of glycolysis- and cholesterol-related genes in each sample, the samples were divided into four subtypes. The samples of glycolysis ≤ 0 and cholesterol ≤ 0 were defined as the quiescent subtype; the samples of glycolysis > 0 and cholesterol ≤ 0 were defined as the glycolysis subtype; the samples of glycolysis ≤ 0 and cholesterol > 0 were defined as the cholesterol subtype; and the samples of glycolysis ≥ 0 and cholesterol ≥ 0 were defined as the mixed subtype (Figure 1B). Detailed information on grouping were presented in Supplementary Table 1. Further analysis of the relationships between the four subgroups and progression-free survival time showed that there were significant differences in prognosis among the four subtypes (*p* < 0.05; Figure 1C). At the same time, there was a significant difference between the cholesterol subtype and quiescent subtype (*p* < 0.05; Figure 1D), There were no significant differences between the other subtypes (Supplementary Figure 2). The expression of glycolysis- and cholesterol-related genes in the four subgroups is shown in Figure 1E, Supplementary Table 6. The expression levels of glycolysis- and cholesterol-related genes were different in the four subtypes.

### Association of Metabolic Subtypes With Molecular Mutations and Copy Number Variations

**Figure 2:**
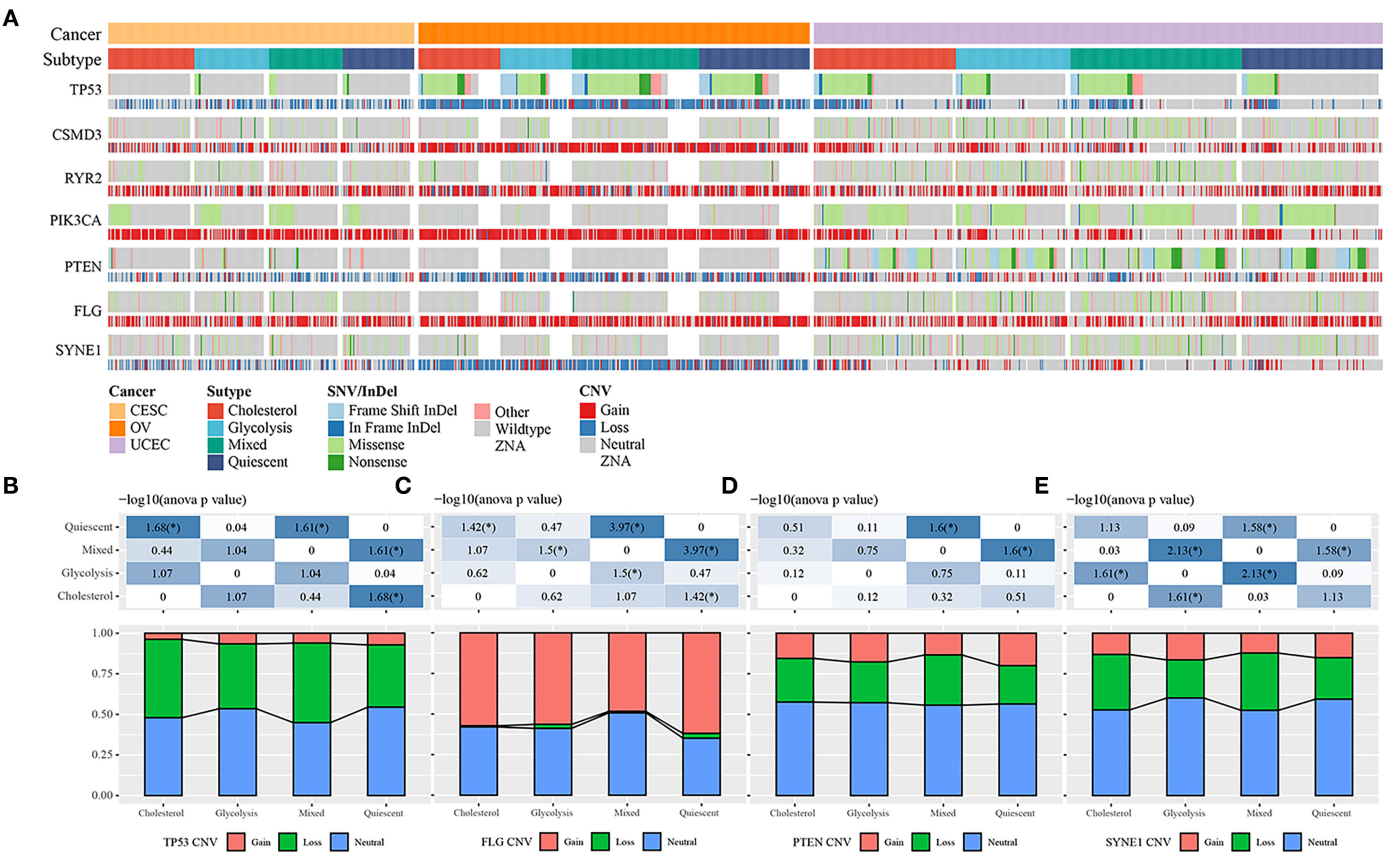
**(A)** Comparison of the distribution of TP53, FLG, PTEN, and SYNE1 in different cancer subtypes. **(B)** The distribution of copy number status of TP53 gene in different subtypes. **(C)** The distribution of the copy number status of the FLG gene in different subtypes. **(D)** Distribution of copy number status of PTEN gene in different subtypes. **(E)** The distribution of copy number status of SYNE1 gene in different subtypes.

Molecular events, such as carcinogenic mutations, including *MYC* amplification, *TP53* mutations, and *PIK3CA* mutations, can drive metabolic reprogramming in cancers, including ovarian, cervical, and endometrial cancer. To determine the carcinogenic events among the different metabolic subtypes, we studied the frequency distribution of mutant genes between the metabolic subtypes affected by SNVs and CNVs (Figure 2A). In the samples with *TP53* loss, the proportion of the cholesterol subtype was significantly higher than that of the quiescent subtype, and in the samples with *TP53* gain, the proportion of the cholesterol subtype was significantly lower than that of the quiescent subtype (Figure 2B). In the samples with *FLG* gain and loss, the proportion of the cholesterol subtype was significantly lower than that of the quiescent subtype (Figure 2C). In the samples with *PTEN* loss, the proportion of the quiescent subtype was significantly higher than that of the mixed group (Figure 2D). In the samples with *SYNE1* loss, the proportion of the glycolysis subtype was significantly lower than that of the mixed group (Figure 2E).

### Comparison of Metabolic Subtypes and Existing Immune Molecular Subtypes

**Figure 3:**
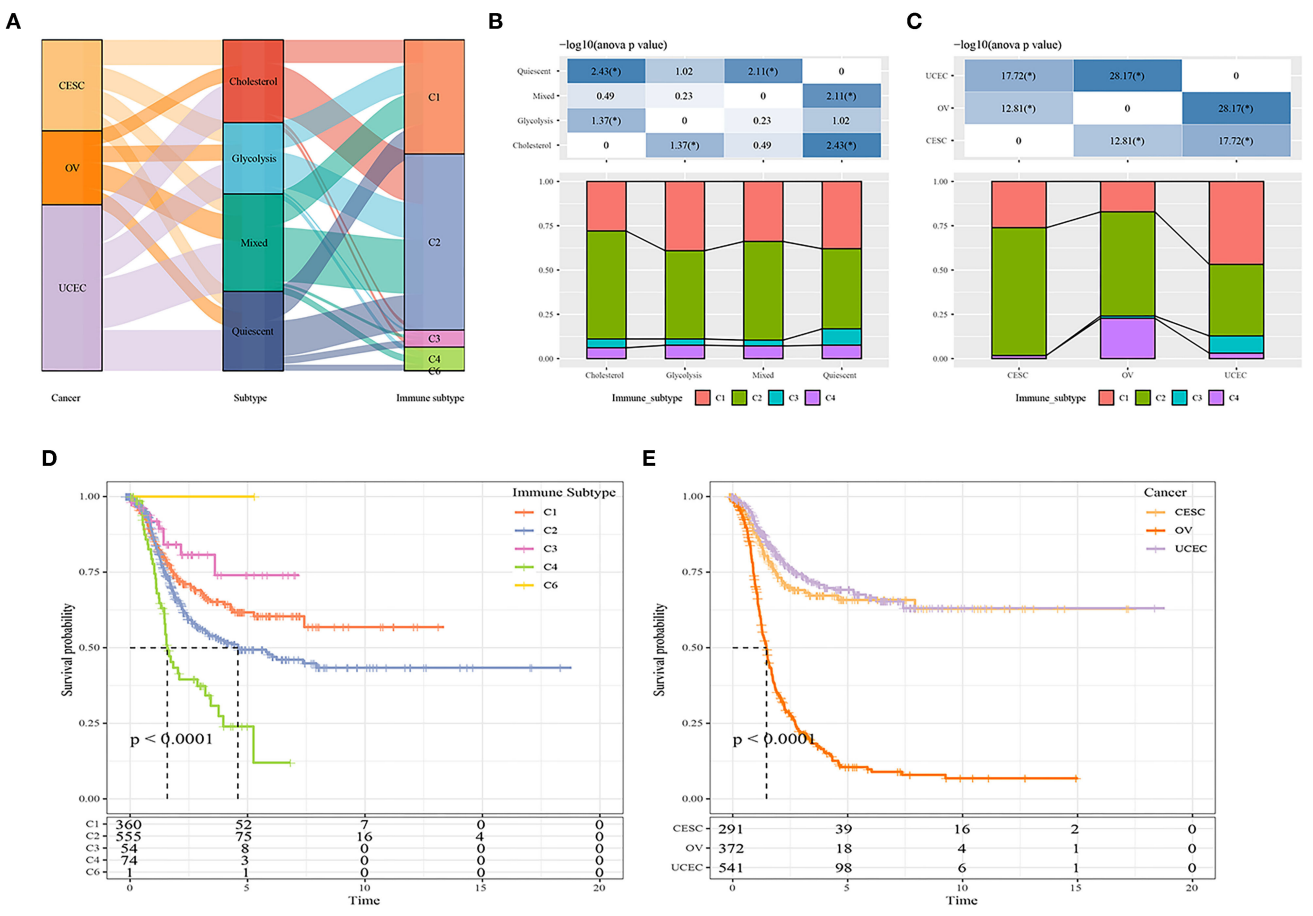
**(A)** Comparison between metabolic subtypes and existing subtypes; **(B)** Comparison of distribution of immune subtypes among different metabolic subtypes; **(C)** Comparison of distribution of immune subtypes among different cancer types. **(D)** The prognostic difference of metabolic molecular subtypes in C1-C6 immune subtypes. **(E)** The prognostic difference of metabolic molecular subtypes in OV, UCEC, CESC tumor types.

Previous studies have classified tumors into six subtypes according to their cellular immune status: C1 (wound healing), C2 (INF-r dominance), C3 (inflammation), C4 (lymphocyte depletion), C5 (immunologically silent), and C6 (transforming growth factor-beta dominance) (15). The majority of ovarian cancer patients in the TCGA database belonged to the C1, C2, and C4 subtypes; the majority of cervical cancer patients belonged to the C1 and C2 subtypes; and the majority of endometrial cancer patients belonged to the C1 and C2 subtypes. The survival curve analysis results showed that there were significant differences in progression-free survival time among the six immune subtypes and OV, UCEC, CESC tumor types (*P* < 0.05; Figures 3D,E). We further compared the distribution among metabolic subtypes, immune subtypes, and tumor types Figures 3A–C, and the results showed that the immune subtypes contained in the cholesterol subtype were significantly different from those in the quiescent subtype. The proportion of the C1 and C3 immune subtypes in the quiescent subtype was higher than in the cholesterol subtype, while the proportion of the C2 immune subtype in the quiescent subtype was lower than in the cholesterol subtype.

### Mitochondrial Pyruvate Carrier Complex as Potential Regulator of Tumor Glycolysis-Cholesterol Synthesis Axis

**Figure 4:**
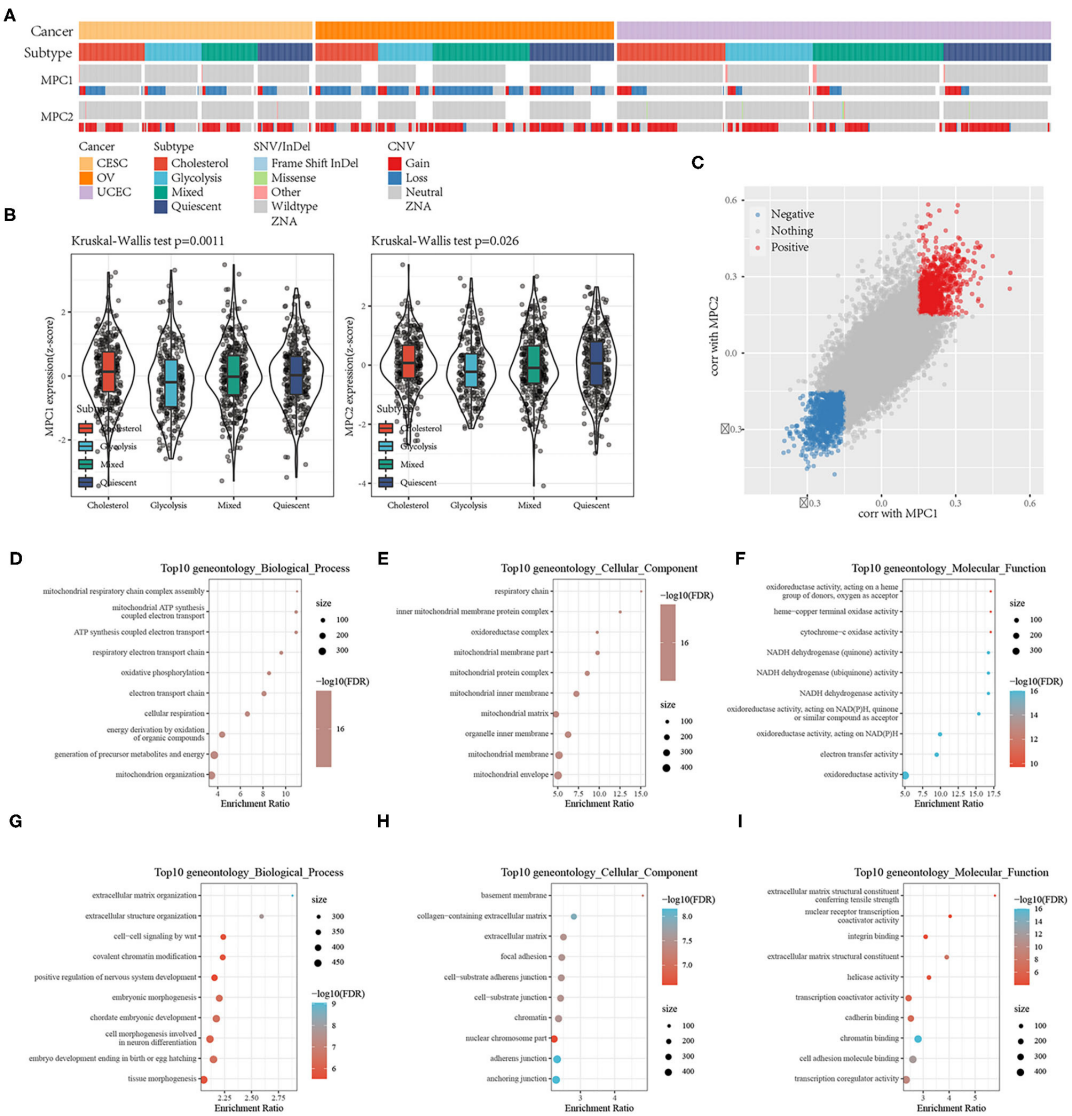
**(A)** Mutations and CNV distribution of MPC1/2 in different metabolic subtypes; **(B)** Comparison of MPC1/MPC2 expression between different metabolic subtypes; **(C)** Scatter plot of MPC1/MPC2 related genes; **(D–F)** GO Functional annotation of genes positively related to MPC1/2; **(G–I)** GO Functional annotation of genes negatively associated with MPC1/2.

The mitochondrial pyruvate carrier (MPC) complex regulates mitochondrial pyruvate flux, inhibits the expression of *MPC1* and *MPC2* in cancer cells, and promotes tumor glycolysis activity and lactate production. To explore the relationships between *MPC1* and *MPC2* expression and glycolysis and cholesterol production, we compared the mutation frequency and expression of these two genes in the metabolic subtypes. There was a contradictory relationship between the two genes in terms of CNV; the CNV that affected *MPC1* was mainly deleted, while the CNV that affected *MPC2* was mostly amplified (Figure 4A). Among the metabolic subtypes, there was a difference in *MPC1* and *MPC2* expression (Figure 4B). The expression levels of *MPC1* and *MPC2* in the Cholesterol group were higher than other subtypes. Study have shown that *MPC* deletion is an effective biomarker of malignant invasion of cancer. Cancer cells re-expressing *MPC1* and *MPC2* will damage their growth characteristics (16), thereby affecting the patient's prognosis.

At the same time, because the expression of *MPC1* and *MPC2* are significantly different among the metabolic subtypes, they may be used as subtype diagnostic markers.

To find pathways related to the expression of *MPC1/2*, we analyzed the correlation between *MPC1/2* and all other genes. A total of 961 genes were positively correlated with *MPC1/2*, and 1,143 genes were negatively correlated with *MPC1/2* (Spearman correlation, FDR < 0.05; Figure 4C). GO functional enrichment analysis of these genes (FDR < 0.05) showed that genes positively related to *MPC1/2* were related to ATP synthesis-coupled electron transport, oxidative phosphorylation, and NADH dehydrogenase (quinone) activity (Figures 4D–F); the genes negatively related to *MPC1/2* were related to extracellular structure organization, cell-cell signaling by wnt, focal adhesion, and transcription coactivator activity (Figures 4G–I). These results suggest that the *MPC1/2* genes are involved in the cell network related to the malignant progression of gynecological pan-cancer.

### Analysis of Differentially Expressed Genes Between Cholesterol and Quiescent Subtypes

**Figure 5:**
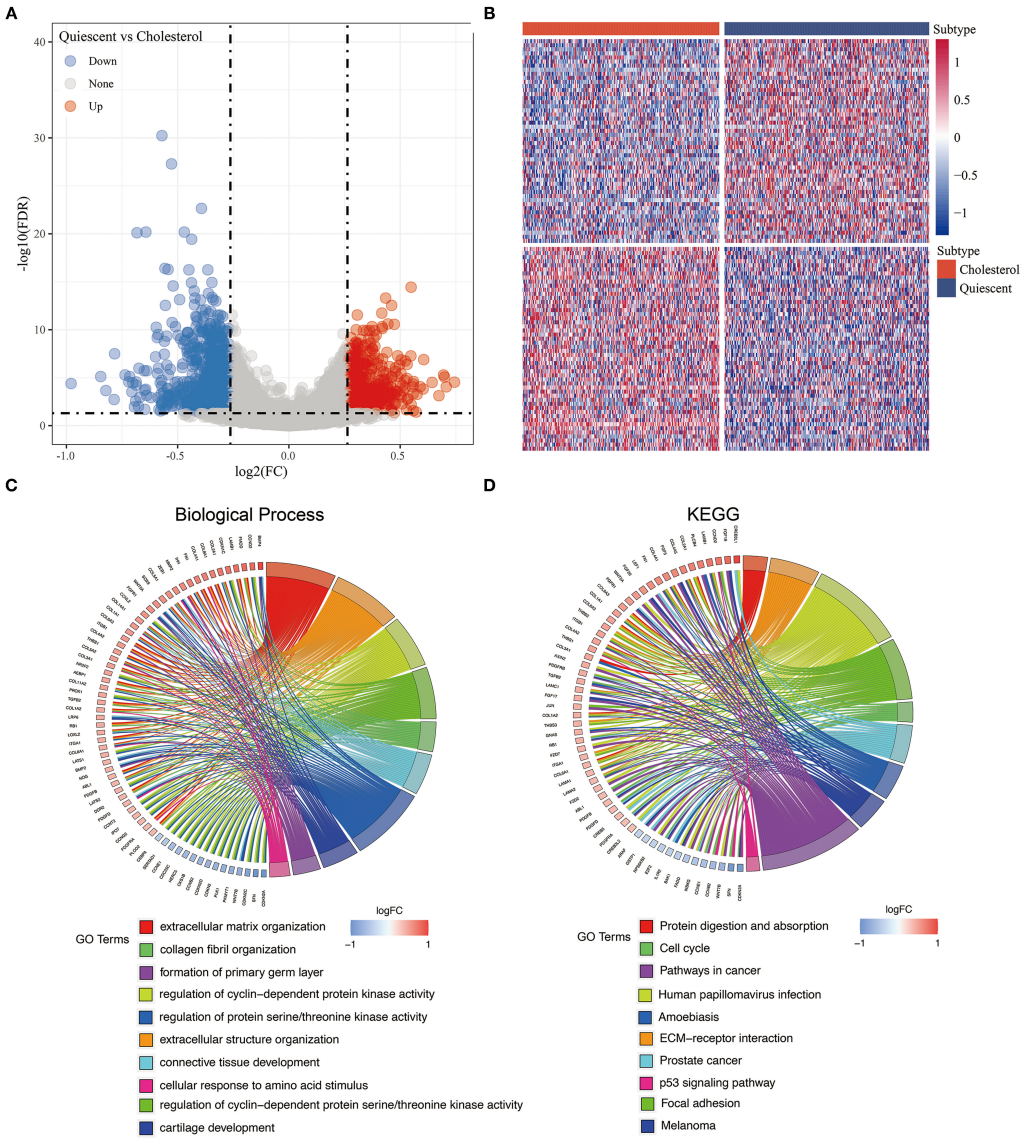
**(A)** The volcano map of the differentially expressed genes in cholesterol and quiescent subtype in the TCGA data set; **(B)** The heatmap of the differentially expressed genes in cholesterol and quiescent subtype in the TCGA data set. **(C)** Biological process enrichment of differentially expressed genes. **(D)** KEGG pathway enrichment of differentially expressed genes.

Among our metabolic subtypes, the prognosis of the cholesterol subtype was the best, while that of the quiescent subtype was the worst, suggesting that for the pan-cancer samples, patients with high expression of cholesterol-related genes had better prognoses, while patients with low expression of glycolysis- and cholesterol-related genes had poor prognoses. To identify the effects of glycolysis- and cholesterol-related gene expression on cancer, we identified the DEGs of the cholesterol and quiescent subtypes and drew a volcano map (Figure 5A, Supplementary Table 7). A total of 1,325 DEGs were obtained, including 562 upregulated genes and 763 downregulated genes. The 100 genes with the largest upregulation and downregulation differences were selected and mapped (Figure 5B). Kyoto Encyclopedia of Genes and Genomes (KEGG) pathway analysis and GO enrichment analysis were performed on the 1,325 DEGs, and the top 10 annotation results were visualized (FDR < 0.05; Figure 5C). The results showed that the p53 signaling pathway, extracellular matrix-receptor interactions, the cell cycle, prostate cancer, focal adhesion, and pathways in cancer were significantly enriched (FDR < 0.05; Figure 5D, Supplementary Table 8).

### Construction and Evaluation of Prognostic Risk Model Based on TCGA-OV

The 372 ovarian cancer samples in the TCGA database were divided into the training set and validation set, with 186 samples in each (Table 1). The training and validation samples were tested by using the chi-square test. The results showed that there was no preference and no significant difference between the groups (*p* > 0.05). Univariable Cox proportional hazard regression was used to analyze the training set, and 12 prognostic genes were obtained (Supplementary Table 9).

**Table 1:** TCGA training set and validation set sample information.

**Clinical features training testing**			***P***
**OS**			
0	48	53	0.641
1	138	133	
**Stage**			
I	0	1	
II	11	10	0.6516
III	141	149	
IV	32	25	
X	2	1	
**Grade**			
G1	1	0	
G2	23	19	0.4086
G3	159	159	
G4	0	1	
GX	3	7	
**Age**			
≤60	96	107	0.2977
>60	90	79	

To further reduce the number of genes, the Akaike information criterion (AIC) was used for stepwise regression. The AIC takes into account both a model's goodness-of-fit and its simplicity in terms of the number of parameters needed to achieve this fit. The stepAIC method in the MASS package started with the most complex model and successively deleted variables to reduce the AIC. The smaller the value, the better the model. It made the model obtain sufficient fit with fewer parameters. Using this algorithm, we eventually reduced the number of genes from 12 to 9.

**Figure 6:**
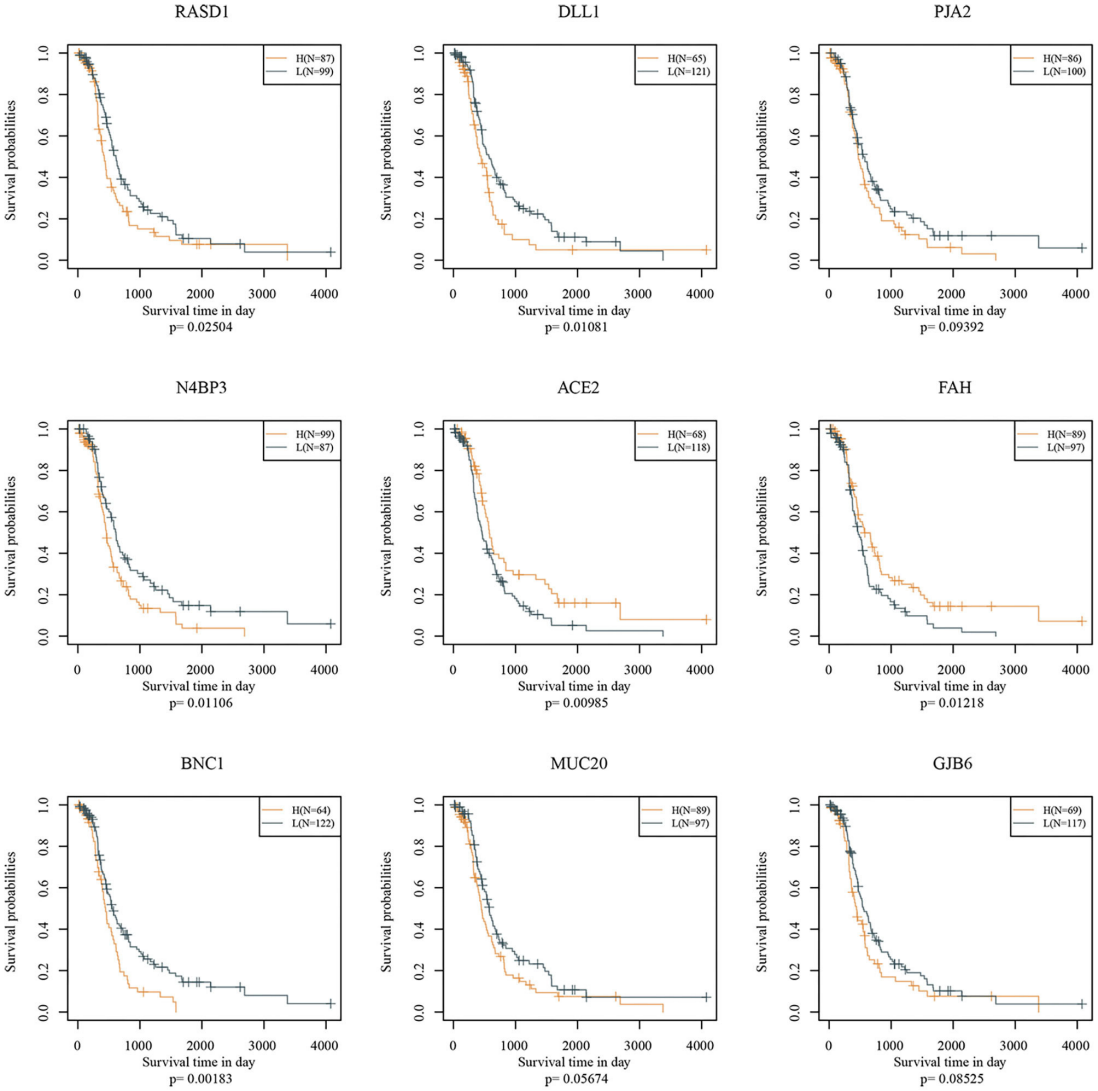
Nine gene KM curves on TCGA training set.

Nine genes, including *RASD1, DLL1, PJA2, N4BP3, ACE2, FAH, BNC1, MUC20*, and *GJB6*, were identified. Prognostic Kaplan-Meier (KM) curves of the nine genes are shown in Figure 6. *PJA2, MUC20*, and *GJB6* could not divide the TCGA training set samples into high- and low-risk groups (*p* > 0.05), while the other genes could. The nine-gene signature formula is as follows: RiskScore = 0.136 ^*^ Rasd1 + 0.124 ^*^ DLL1 + 0.242 ^*^ PJA2 + 0.21 ^*^ N4BP3 − 0.231 ^*^ ACE2 − 0.297 ^*^ FAH + 0.22 ^*^ BNC1 + 0.143 ^*^ MUC20 + 0.082 ^*^ GJB6.

**Figure 7:**
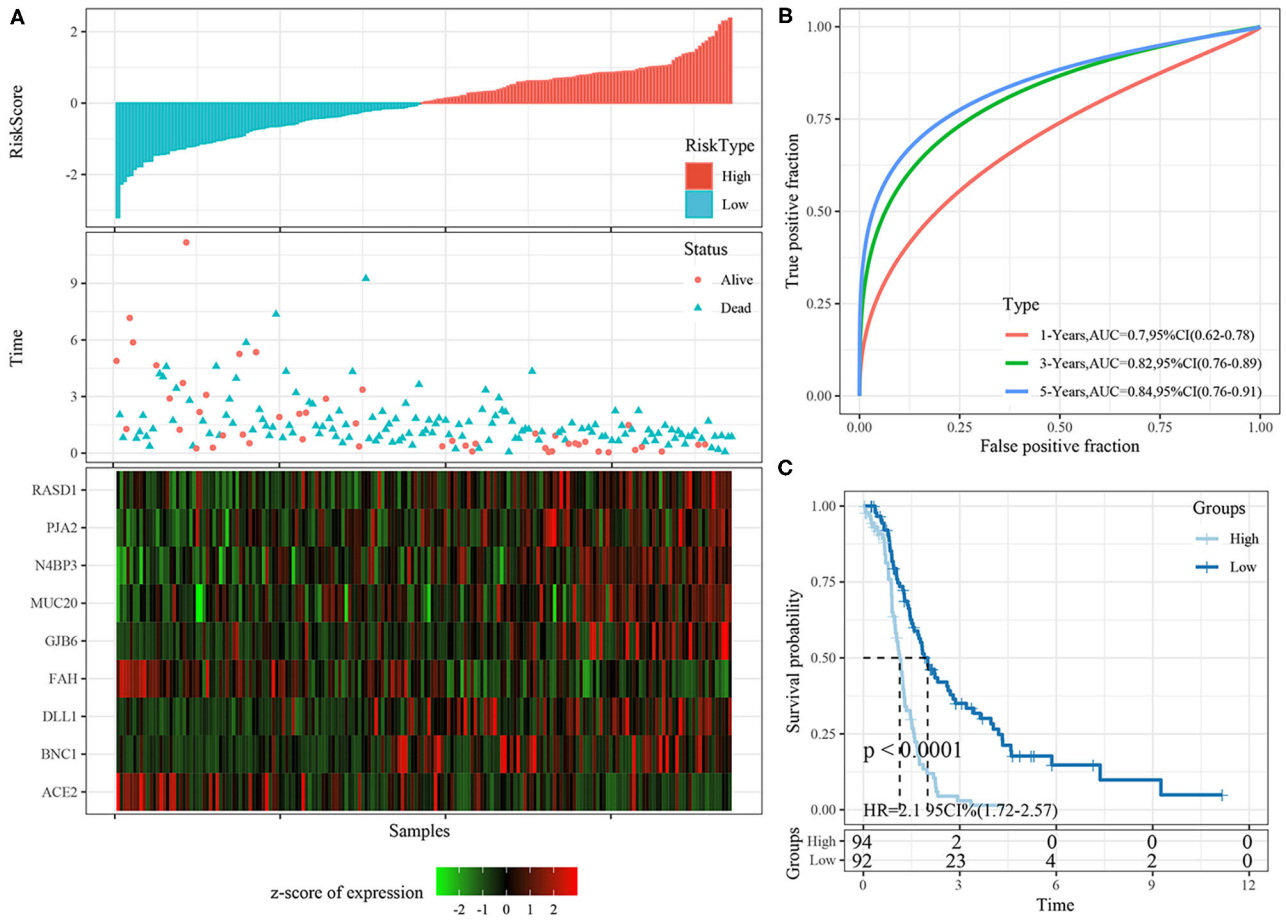
**(A)** The distribution of risk score, survival time, and the expression of 9-gene in TCGA training cohort; **(B)** The ROC curve of 9-gene signature in training cohort; **(C)** The KM survival curve of 9-gene signature in training cohort.

Risk scores were calculated for each sample based on their gene expression levels, and the risk score distribution was plotted (Figure 7A). The samples with high risk scores had significantly shorter survival times than the samples with low scores, suggesting that the samples with high risk scores had worse prognoses. The 1-, 3-, and 5-year predictive classification efficiency was assessed (Figure 7B). Risk scores greater than zero were divided into the high-risk group (94 samples), and those less than zero were divided into the low-risk group (92 samples). The KM survival curve showed a significant difference between the high- and low-risk groups (*p* < 0.0001; Figure 7C).

### Robustness of Nine-Gene Signature in Internal Validation Cohorts

**Figure 8:**
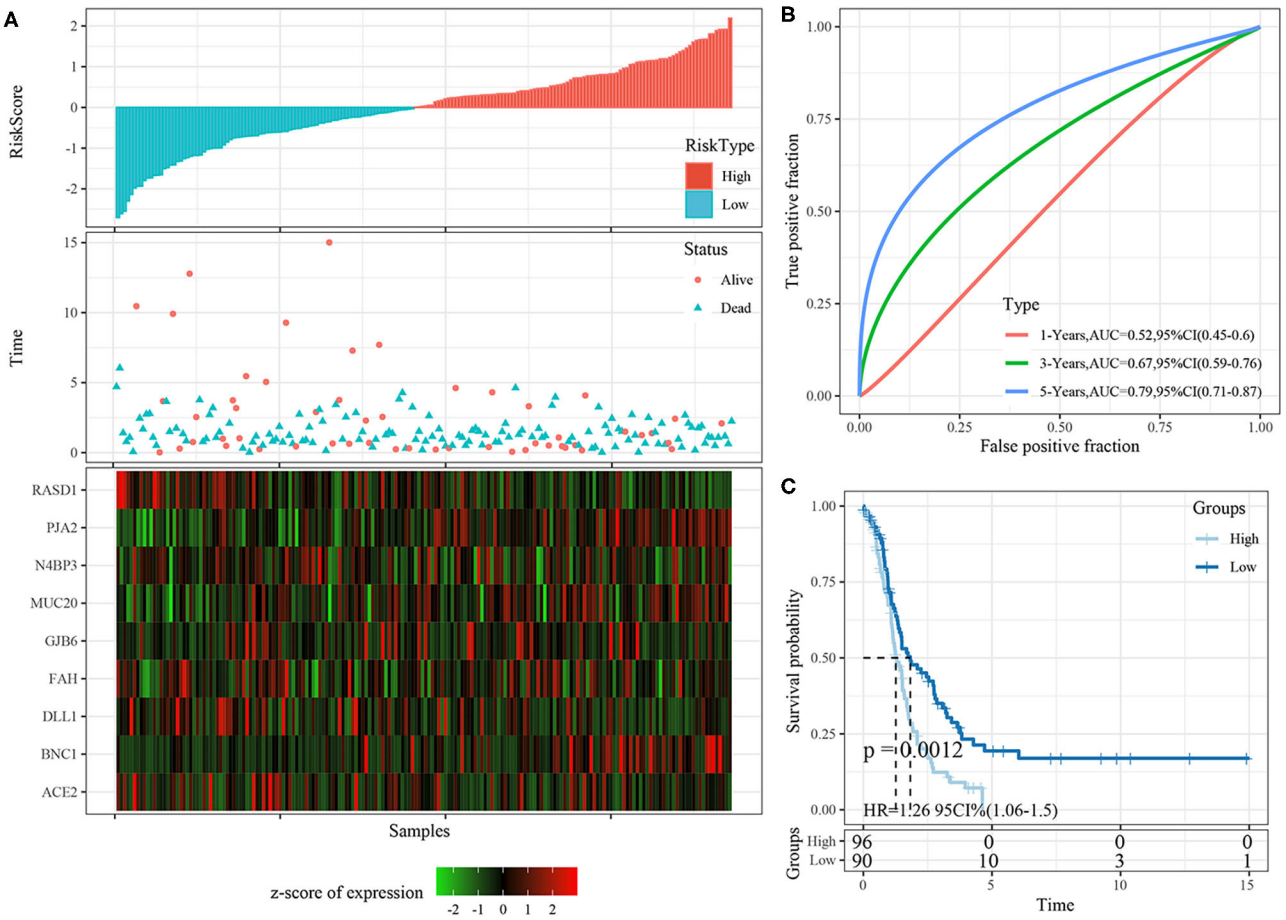
**(A)** The distribution of risk score, survival time, and the expression of 9-gene in TCGA testing cohort; **(B)** The ROC curve and AUC curve of 9-gene signature in testing cohort; **(C)** The KM survival curve of 9-gene signature in testing cohort.

**Figure 9:**
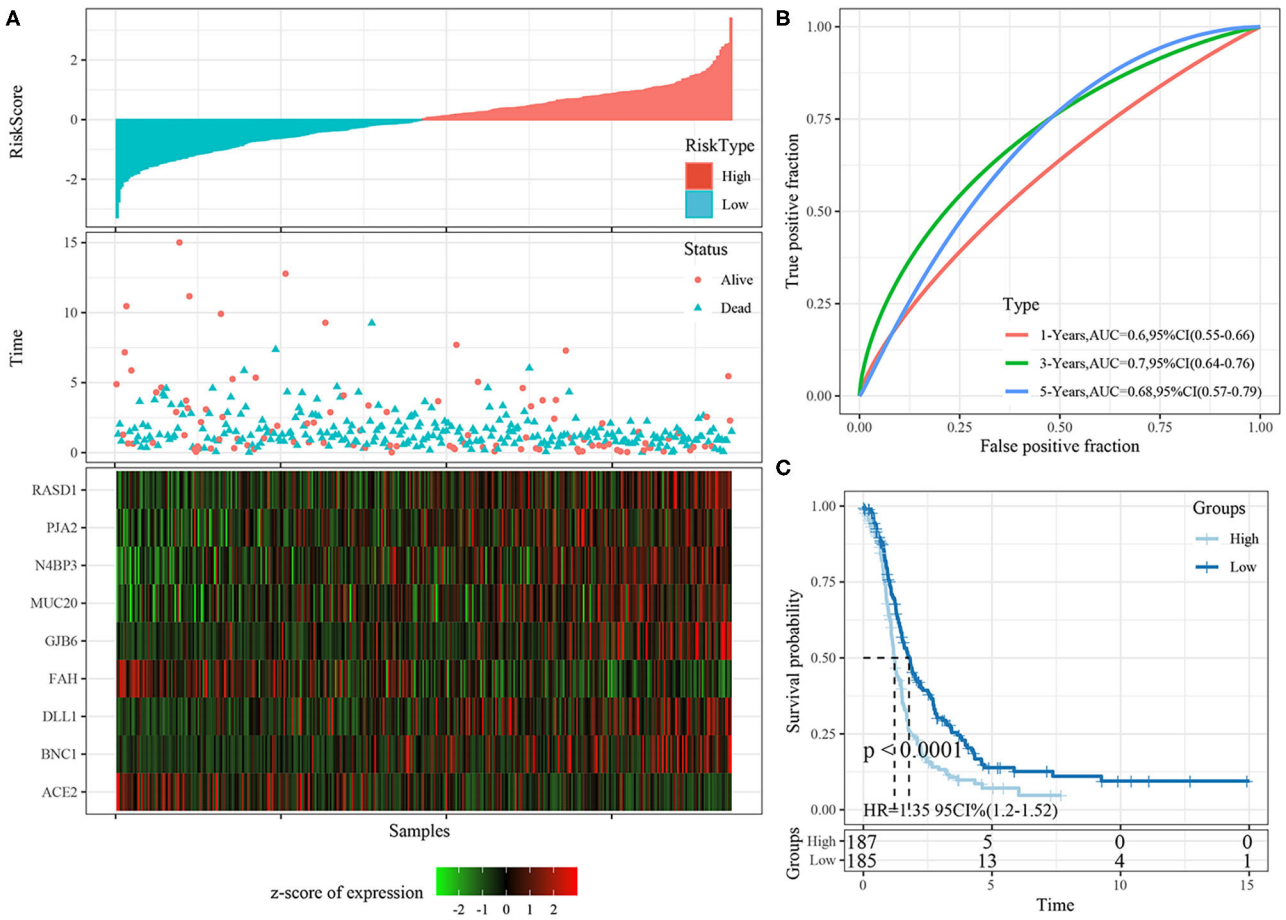
**(A)** The distribution of risk score, survival time, and the expression of 9-gene in TCGA-OV cohort; **(B)** The ROC curve and AUC curve of 9-gene signature in TCGA-OV cohort; **(C)** The KM survival curve of 9-gene signature in TCGA-OV cohort.

The risk score distributions of the internal validation cohorts (testing cohort, all TCGA cohort), are shown in Figures 8A, 9A, respectively. The ovarian cancer samples with high risk scores had shorter survival times than those with low risk scores, which is consistent with the trend of the training cohort, suggesting that samples with high risk scores had worse prognoses. The predictive classification efficiencies at 1, 3, and 5 years are shown in Figures 8B, 9B, respectively. The 5-year area under the curve reached 0.79 in the testing cohort, 0.68 in all TCGA cohort.

Risk scores greater than zero were divided into the high-risk group, and those less than zero were divided into the low-risk group. In the testing cohort, 96 samples were divided into the high-risk group and 90 into the low-risk group. In all TCGA cohort, 187 samples were classified into the high-risk group and 185 into the low-risk group. The KM survival curve showed significant differences between the high- and low-risk groups in internal validation cohorts (Figures 8C, 9C, *p* < 0.05).

### Robustness of Nine-Gene Signature in External Validation Cohort

**Figure 10:**
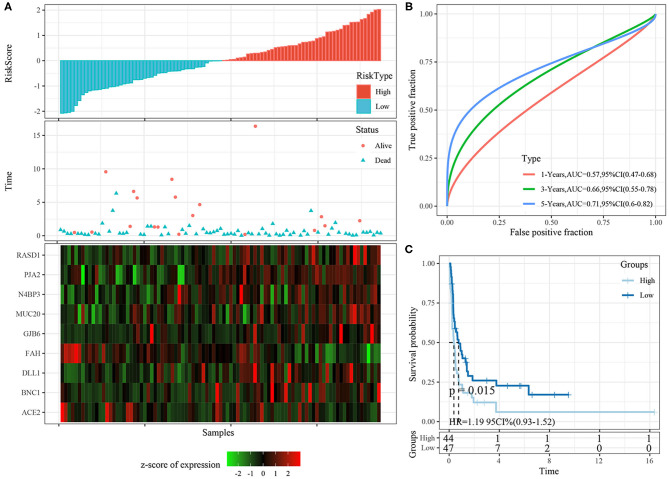
**(A)** The distribution of risk score, survival time, and the expression of 9-gene in ICGC cohort; **(B)** The ROC curve and AUC curve of 9-gene signature in ICGC cohort; **(C)** The KM survival curve of 9-gene signature in ICGC cohort.

The risk score distributions of the ICGC cohort was shown in Figure 10A. The samples with high risk scores had worse prognose than those with low risk scores, which is consistent with the trend of the training cohort. The predictive classification efficiencies at 1, 3, and 5 years are shown in Figure 10B. The 5-year area under the curve reached 0.71.

Risk scores greater than zero were divided into the high-risk group, and those less than zero were divided into the low-risk group. In the ICGC datasets, 44 samples were divided into the high-risk group and 47 into the low-risk group. The KM survival curve showed significant differences between the high- and low-risk groups (Figure 10C, *p* < 0.05).

### Correlation Analysis Between Risk Model and Clinical Features

**Figure 11:**
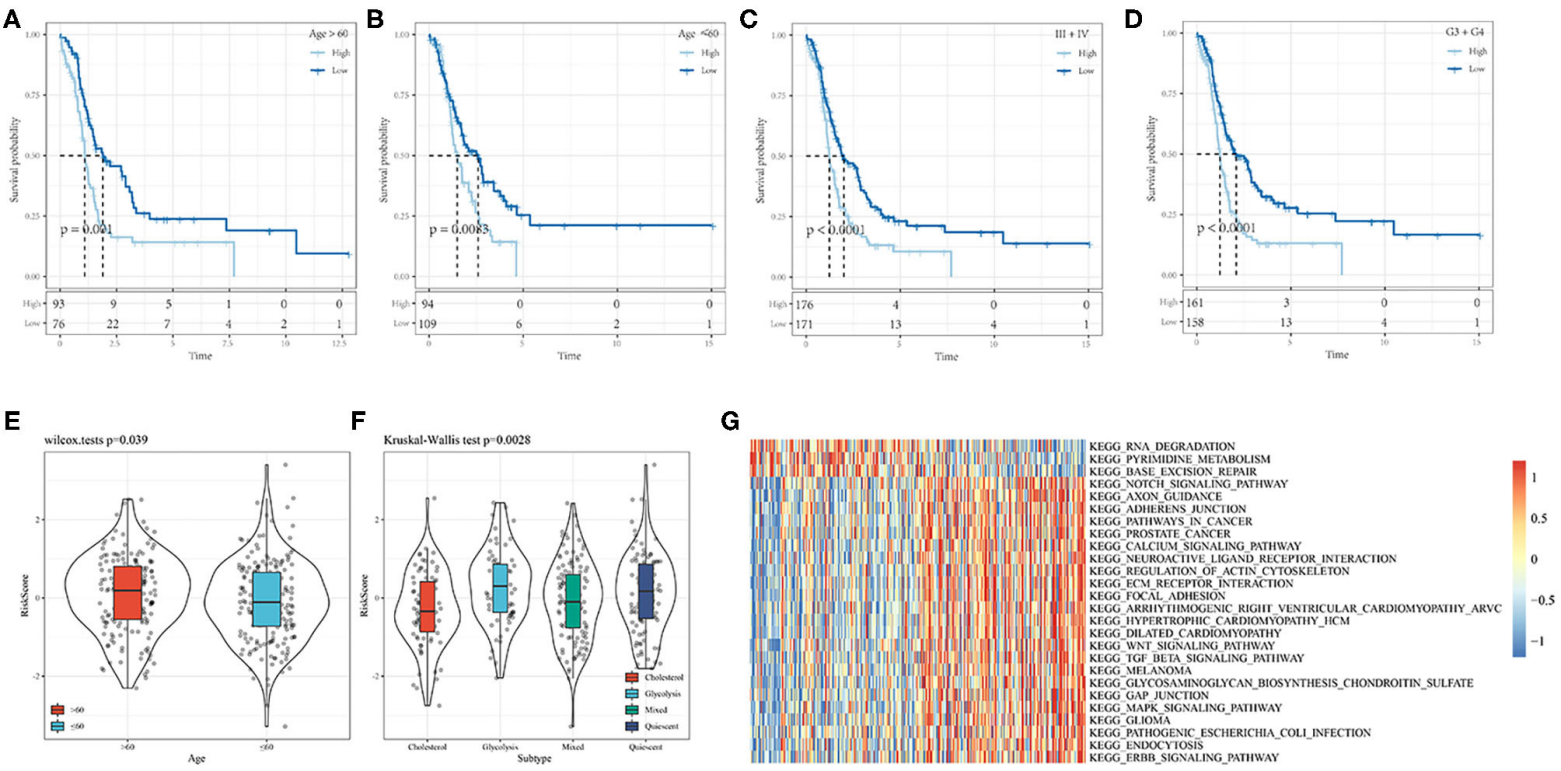
**(A,B)** Comparison of prognosis of different Age groups based on Riskscore. **(C)** Comparison of prognosis of Stage III-IV based on Riskscore; **(D)** Comparison of prognosis of Grade 3+4 based on Riskscore; **(E)** Correaltion of RiskScore between samples in Age groups; **(F)** Correaltion of RiskScore between samples in molecular subtype groups; **(G)** Heat map of the relationship between risk score and biological function.

Based on the nine-gene signature risk score, age, stage, and grade could be significantly divided into high- and low-risk groups with different prognoses (*p* < 0.05; Figures 11A–D). The nine-gene signature model thus had good predictive ability in terms of different clinical features. The risk score was significantly correlated with the clinical feature score, and the risk score of the older group was significantly higher than that of the younger group. Regarding the subtypes, the risk score of the quiescent subtype with poor prognosis was higher, while the risk score of the cholesterol subtype with good prognosis was lower (*p* < 0.05; Figures 11E,F).

The relationships between the risk scores and biological functions of 372 ovarian cancer samples (Supplementary Table 10) were further analyzed by gene set enrichment analysis, and the first 26 KEGG pathways with correlations greater than 0.35 were selected. Cluster analysis results based on the enrichment scores are shown in Figure 11G. It can be seen that tumor-related pathways, including KEGG_NOTCH_SIGNALING_PATHWAY KEGG_WNT_ SIGNALING_ PATHWAY, KEGG_TGF_ BETA_ SIGNALING_ PATHWAY, KEGG_ FOCAL_ ADHESION, and KEGG_MAPK_ SIGNALING_ PATHWAY activated with increased risk score.

### Prognostic Independence Analysis of Risk Signature

**Figure 12:**
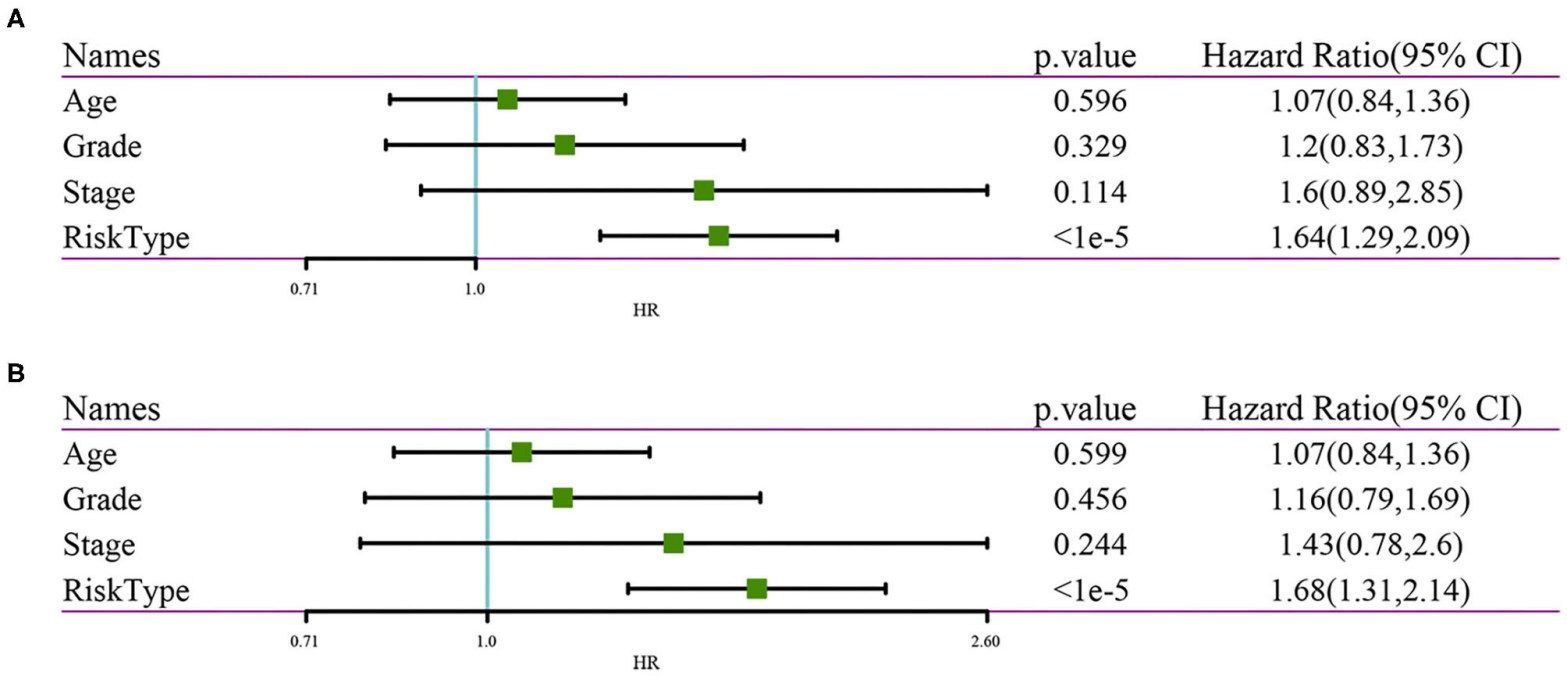
**(A)** Clinical features and results of univariate analysis by RiskScore; **(B)** Clinical Features and results of multivariate analysis by RiskScore.

Both univariate (HR = 1.64, *p* < 1e−5) and multivariate Cox regression analyses (HR = 1.68, *p* < 1e−5) showed that the nine-gene signature was significantly associated with prognosis. However, age, grade, stage were not significant in both univariate and multivariate Cox regression analyses. This further showed that in predicting the prognosis of patients with ovarian cancer, the nine-gene signature had better performance comparing with other clinical variables (Figures 12A,B).

### Comparison of Risk Model With Other Models

Three published prognosis-related risk models were selected for comparison with our nine-gene signature: a nine-gene signature (17), a seven-gene signature (18), and a five-gene signature (19). Kaplan Meier curves and ROC curve were performed to make models comparable. First, we used the same method to calculate every risk score in the TCGA data according to the corresponding genes in the three models, then the Z-score of the risk score was calculated, and the risk scores were divided into high- and low-risk groups.

**Figure 13:**
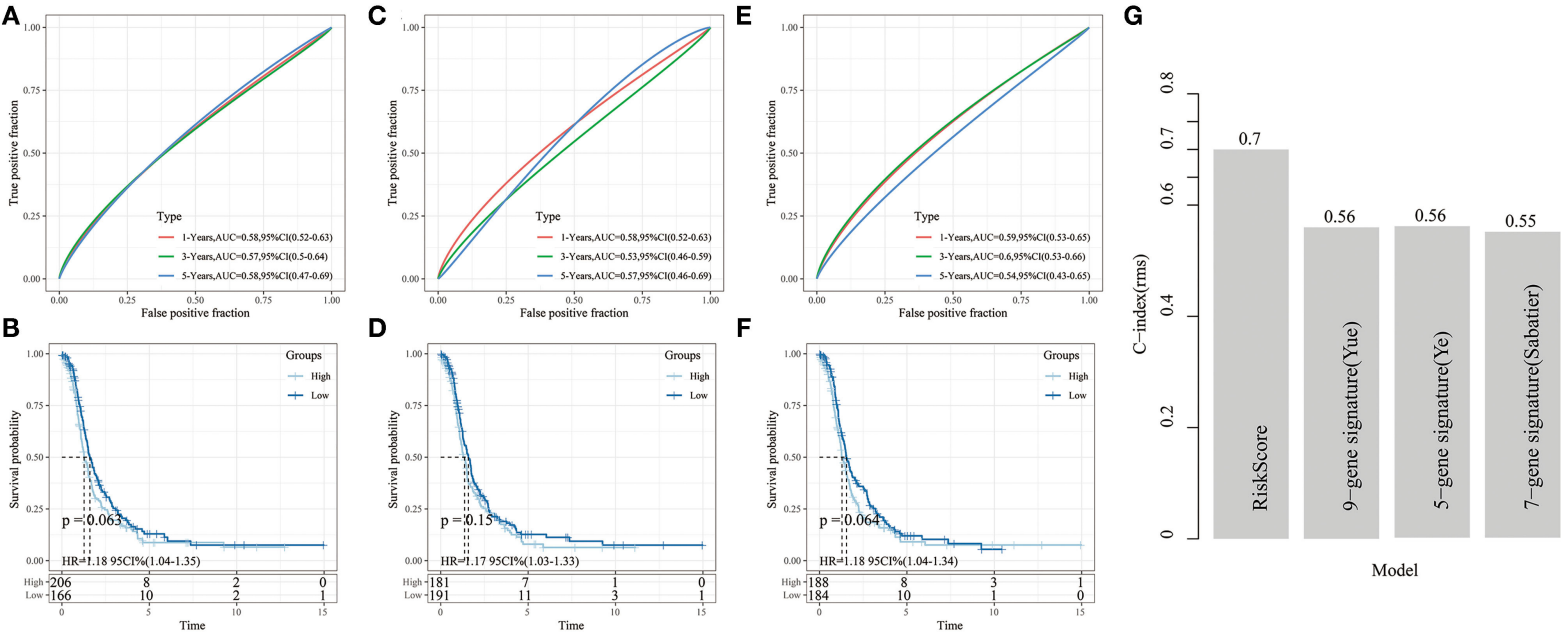
**(A,B)** ROC analysis of 9-gene signature (Yue) and KM curve of High/Low samples; **(C,D)** ROC analysis of 7-gene signature (Sabatier) and KM curve of High/Low samples; **(E,F)** ROC analysis of 5-gene signature (Ye) and KM curve of High/Low grouping sample. **(G)** The C-index comparison curve among four prognostic signature.

The KM survival curve showed no significant prognostic differences between the high- and low-risk groups between these datasets (Figures 13B,D,F). Moreover, the ROC curve at 1, 3, and 5 years in the three models were all lower than in our signature (Figures 13A,C,E).

Furthermore, to compare the prediction performance of these models in ovarian cancer more intuitive, we used the *rms* package in R to calculate the concordance index (C-index) of the different models. We calculated the C-index of the 4 models in the TCGA cohort. It showed that our signature had a significantly higher average C-index than the other models (Figure 13G), indicating that the overall performance of our gene signature outweighed that of the other 3 signatures.

## Discussion

Epithelial ovarian cancer is one of the deadliest gynecological malignancies in the western world. Most patients with advanced epithelial ovarian cancer develop recurrence and chemotherapy resistance (20, 21). There are many metabolic pathways in tumor cells, such as fatty acid, glutamine, serine, and cholesterol metabolism (22). Aerobic glycolysis is closely related to tumor growth and chemotherapy resistance (23–25). Previous study has shown that the tumorigenicity of epithelial ovarian cancer cells depends on their glycolytic phenotype and that cells with a greater glycolytic phenotype are more aggressive (26). Meanwhile, studies have shown that the metabolites involved in fatty acid metabolism are increased in both primary and metastatic ovarian cancer (27). Changes in lipid metabolism can lead to increased proliferation, invasion, and migration of cancer cells, resulting in tumor metastasis (28). There is increasing evidence that obesity is a significant risk factor for ovarian cancer, and elevated low-density lipoprotein cholesterol levels, which are a common complication of obesity, may also indicate poor prognoses in patients with ovarian cancer (29–31). Pyruvic acid is an intermediate metabolite of the tricarboxylic acid cycle and provides the precursor citrate for fat formation, including the biosynthesis of cholesterol and free fatty acids. The activation of the mevalonate pathway induced by oncogenes is very important for the synthesis of cholesterol from scratch. Simvastatin belongs to the family of statins and is widely used in the treatment of hypercholesterolemia. Statins inhibit 3-hydroxy-3-methyl-glutaryl-coenzyme A reductase, which is essential for the synthesis of mevalonate (32). Studies have shown that long-term use of statin derivatives, such as cholesterol synthesis inhibitors, can improve the prognoses of patients with ovarian cancer (33, 34). Therefore, metabolic pathways play an important role in the malignant progression and targeted therapy of tumors. Thus, it is of great significance to elucidate the metabolic pathways of gynecological malignant tumors for their prevention and treatment.

In this study, 46 cholesterol- and glycolysis-related genes were used for gene clustering, and 11 glycolytic co-cluster genes and seven cholesterol co-cluster genes were obtained. The TCGA data including ovarian, endometrial, and cervical cancer samples were divided into four subtypes: quiescent, glycolysis, cholesterol, and mixed. The survival analysis results showed that the prognosis of the cholesterol subtype was better than that of the quiescent subtype, suggesting that the prognoses of patients with high expression of cholesterol-related genes were better than those with low expression of cholesterol- and glycolysis-related genes. These results suggested the presence of metabolic phenotypes related to glycolysis, cholesterol production, and prognosis in gynecological pan-cancer. By comparing the mutations and copy number variations among the four subtypes, we found that the proportion of the cholesterol subtype was higher than that of the quiescent subtype in the *TP53* loss samples, and in the samples with *FLG* gain, the proportion of the cholesterol subtype was lower than that of the quiescent subtype. These results suggested that abnormal expression of *TP53* and *FLG* could promote the malignant progression of tumors by promoting cholesterol synthesis and changing cholesterol metabolism.

Pyruvate is the hub of carbohydrate, fat, and amino acid metabolism, and the overall metabolic state of cells determines the metabolism of pyruvate. Under aerobic conditions, a carrier transports pyruvate from the cell matrix to the mitochondrial matrix, and it is then converted by the pyruvate dehydrogenase complex into acetyl-coenzyme A and carbon dioxide. Under anaerobic conditions, the glycolysis of pyruvic acid in cytoplasm produces lactic acid, which is transferred from the cell. MPC is a protein that plays an important role in the passage of pyruvate through the mitochondrial membrane. MPC consists of MPC1 and MPC2 subunits (35–37). Inactivation of any of these subunits leads to the loss of activity of the MPC complex and a decrease in mitochondrial pyruvate transport and utilization (38). MPC deficiency can lead to metabolic disorders and changes in tumor metabolism (16, 39, 40). In most tumor cells, MPC1 and MPC2 levels are low or not present, and patients with low MPC1 levels usually have poor prognoses (41). The expression of *MPC1/2* in different metabolic subtypes was also analyzed in this study. The results showed that there were significant differences in *MPC1/2* expression among different metabolic subtypes, suggesting that abnormal changes in MPC complex regulation of pyruvate flux may participate in the malignant progression of gynecological pan-cancer by affecting metabolic pathways.

We focused on analyzing the cholesterol subtype with good prognosis and the quiescent subtype with poor prognosis. We identified DEGs between the two subtypes and conducted functional enrichment analysis. The results showed that DEGs between the two subtypes were significantly enriched in the p53 signaling pathway, extracellular matrix-receptor interactions, the cell cycle, prostate cancer, focal adhesion, and pathways in cancer, suggesting that the cholesterol subtype may affect the malignant progression of gynecological pan-cancer through the above pathways. In addition, there was a significant correlation between tumor-related transforming growth factor and the quiescent subtype, and there were also correlations between the cholesterol subtype and metabolism pathways, such as CHOLESTEROL_HOMEOSTASIS and FATTY_ACID_METABOLISM. Cholesterol homeostasis plays an important role in the progression of cancer. In ovarian cancer, cholesterol homeostasis may modulate the sensitivity of ovarian cancer patients to platinum-based drugs (42). Our results suggested that cholesterol-related genes may be involved in drug resistance through cholesterol homeostasis, fatty acid metabolism, and other pathways in gynecological pan-cancer, but these are rarely studied in cervical and endometrial cancers.

Based on the DEGs of the cholesterol and quiescent subtypes, we constructed a nine-gene signature prognostic model including *Rasd1, DLL1, PJA2, N4BP3, ACE2, FAH, BNC1, MUC20*, and *GJB6* in the ovarian cancer samples, and we compared it with existing prognostic models [a nine-gene signature (17), a seven-gene signature (18), and a five-gene signature (19)]. Our model was robust in both the training and verification datasets, and it also had excellent prediction performance.

## Conclusion

In conclusion, the metabolic classification of gynecological pan-cancer based on metabolic reprogramming may provide an important basis for clinicians to choose treatment options, predict treatment resistance, and predict patients' clinical outcomes.

## Data Availability Statement

The original contributions presented in the study are included in the article/Supplementary Material, further inquiries can be directed to the corresponding author/s.

## Author Contributions

GW designed and conceived the current study. XL collected and conducted the analyses. GW and XL wrote the initial draft of the paper. DW and MS edited and interpreted the manuscript. QY supervised the study. All authors read and approved the final version of the manuscript and agreed to be accountable for all aspects of the research in ensuring that the accuracy or integrity of any part of the work are appropriately investigated and resolved.

## Conflict of Interest

The authors declare that the research was conducted in the absence of any commercial or financial relationships that could be construed as a potential conflict of interest.
