# Supplementary figures and images for: Identification and Development of Subtypes With Poor Prognosis in Pan-Gynecological Cancer Based on Gene Expression in the Glycolysis-Cholesterol Synthesis Axis
Source: Front Oncol. 2021 Mar 24;11:636565. doi: 10.3389/fonc.2021.636565 (PMC8025671; doi:10.3389/fonc.2021.636565)

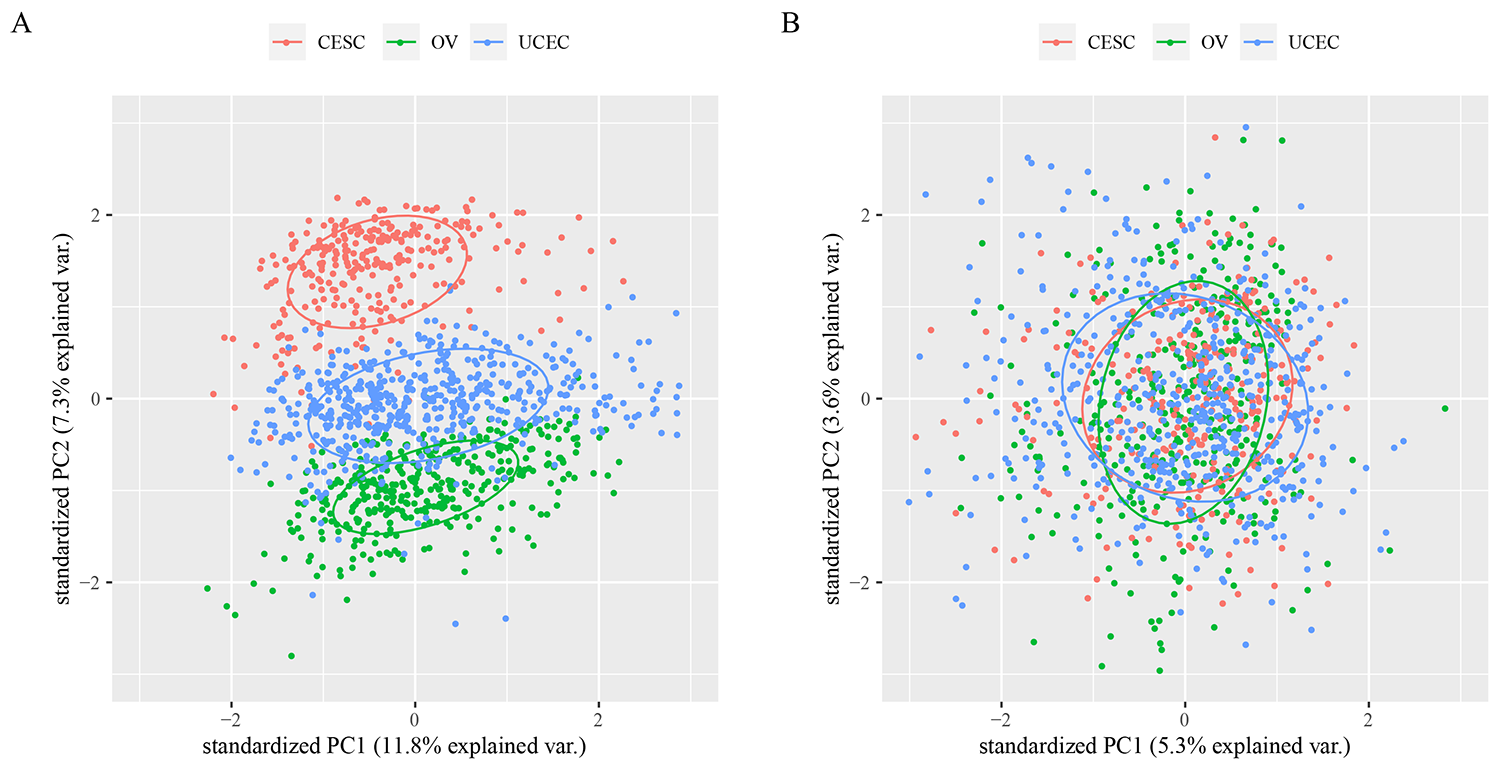

Supplement: Supplementary file 11 [file Image_1.TIF]

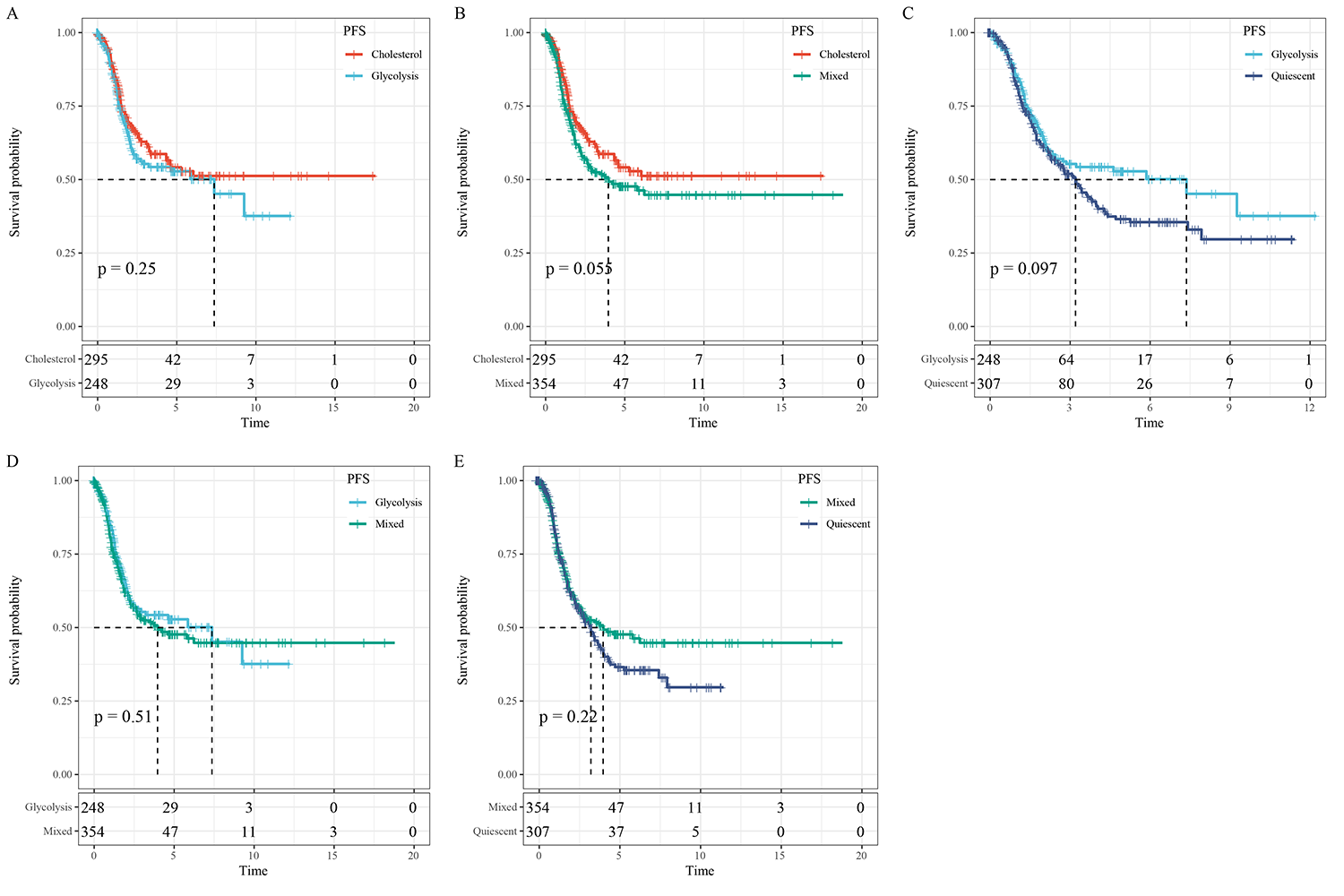

Supplement: Supplementary file 12 [file Image_2.TIF]
